# Supplementary material for: Evaluation of the Synthetic Multifunctional Peptide Hp-MAP3 Derivative of Temporin-PTa
Source: Toxins (Basel). 2023 Jan 5;15(1):42. doi: 10.3390/toxins15010042 (PMC9866994; doi:10.3390/toxins15010042)
Supplement: Supplementary file 1 [file toxins-15-00042-s001.zip › toxins-1884032-supplementary.pdf]

Supplementary Materials: Evaluation of the Synthetic Multifunctional Peptide Hp-MAP3 Derivative of Temporin-PTa

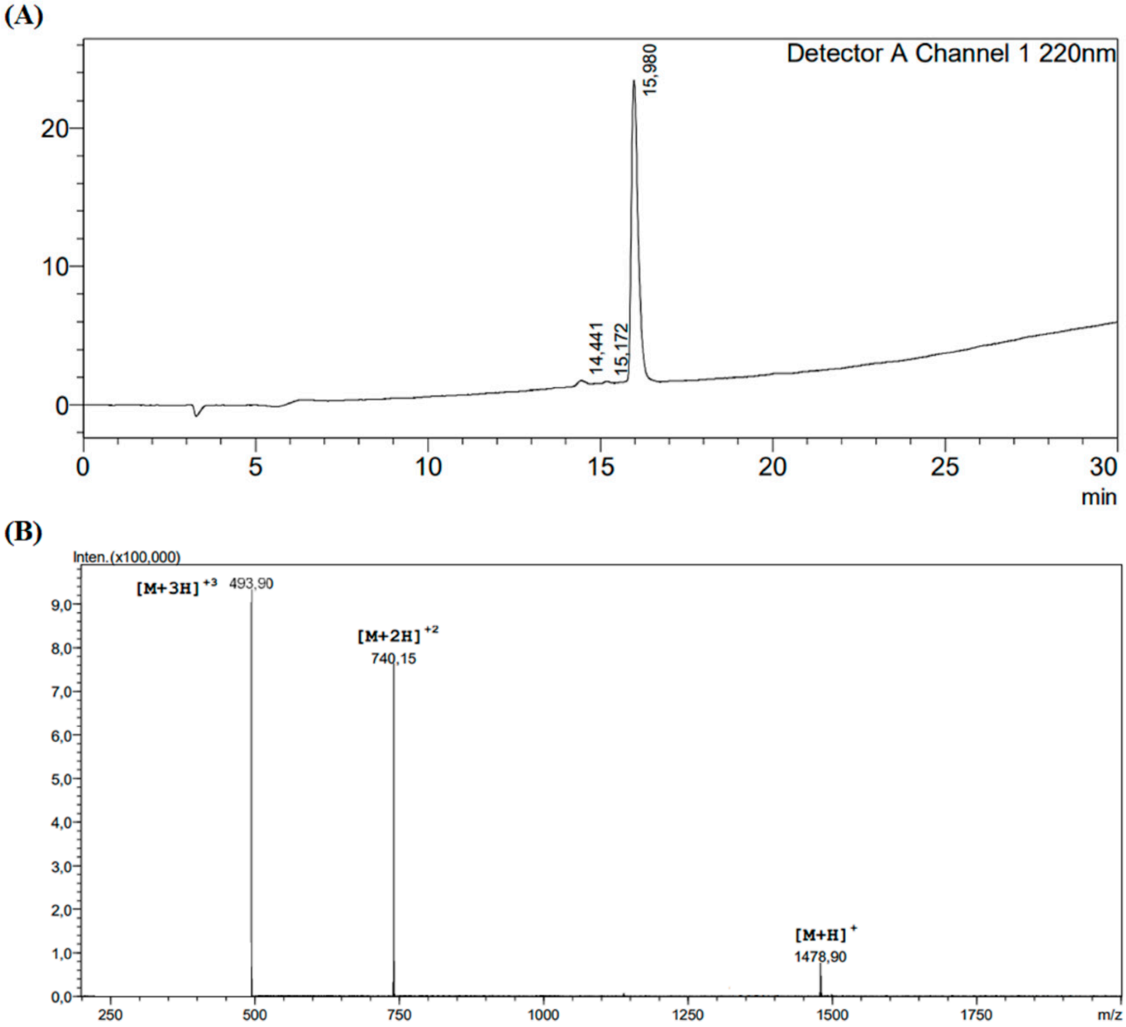

Figura S1: Purification and confirmation of the molecular mass of the Hp-MAP3 peptide. **(A)** Profile Purification by reversed phase high performance liquid chromatography (RP-HPLC) to >95% purity. **(B)** Electrospray ionization mass spectrometry (ESI-MS) The profile represents monoisotopic masses $[M+H]^+$ of 1.478 Da respectively.

Table S1: Antibiogram assay against certain bacteria. (+) resistant at the concentrations tested.

**Microorganism**	Amoxicillin	Imipenem	Erythromycin	Ampicillin
**Gram-negative**	**>350.3 (μM)**	**>427.6 (μM)**	**>174.4 (μM)**	**>366.3 (μM)**
Acinetobacter baumannii (clinical isolated)	+	+	+	+
Escherichia coli (clinical isolated)	+	+	+	+
Escherichia coli (KPC)	+	+	+	+
Klebsiella pneumoniae (ATCC)	+	+	+	+
Klebsiella pneumoniae (KPC)	+	+	+	+
Pseudomonas aeruginosa (ATCC)	+	+	+	+
**Gram-positive**				
Staphylococcus aureus (clinical isolated)	+	+	+	+
